# New Appliances & Things Medical

**Published:** 1908-11-07

**Authors:** 


### NEW APPLIANCES & THINGS MEDICAL.

### HYGIENIC PAPER HANDKERCHIEFS.

We have drawn attention before now to the many advantages attending the use of pocket-handkerchiefs which can be destroyed after use. In winter time especially, the need is felt for some hygienic and convenient substitute for the ordinary linen handkerchief. Tubercle bacilli and the micro-organisms of influenza and of coryza, for example, are undoubtedly disseminated by the usual practice of replacing the handkerchief saturated with infected secretions in the pocket. The handkerchief is then sent to the laundry, and mixed up with other articles in the washing, and the contaminated pocket remains ready to infect the next clean handkerchief which it receives. By the use of *Papkerchiefs*, specially prepared squares of soft crêpe paper, which can be carried in a special flat holder and burnt after use, these risks of infection are minimised. *Papkerchiefs* are manufactured from vegetable fibre, and are light, unirritating, and absorbent. Their price (from 3s. 6d. per 1,000) is within the reach of all; and they can be recommended with confidence. Their widespread use would certainly be in the interests of the public health. The address of the manufacturers is 99-101 Kingsland Road, London, N.E.

